# Heparan Sulfate and Heparanase as Modulators of Breast Cancer Progression

**DOI:** 10.1155/2013/852093

**Published:** 2013-07-31

**Authors:** Angélica M. Gomes, Mariana P. Stelling, Mauro S. G. Pavão

**Affiliations:** ^1^Programa de Glicobiologia, Instituto de Bioquímica de Médica, Universidade Federal do Rio de Janeiro, Rio de Janeiro, RJ 21941-590, Brazil; ^2^Laboratório de Bioquímica e Biologia Celular de Glicoconjugados, Hospital Universitário Clementino Fraga Filho, Rua Prof. Rodolpho P. Rocco No. 255, 4 andar, sala 4A-08, Rio de Janeiro, RJ 21941-590, Brazil

## Abstract

Breast cancer is defined as a cancer originating in tissues of the breast, frequently in ducts and lobules. During the last 30 years, studies to understand the biology and to treat breast tumor improved patients' survival rates. These studies have focused on genetic components involved in tumor progression and on tumor microenvironment. Heparan sulfate proteoglycans (HSPGs) are involved in cell signaling, adhesion, extracellular matrix assembly, and growth factors storage. As a central molecule, HSPG regulates cell behavior and tumor progression. HS accompanied by its glycosaminoglycan counterparts regulates tissue homeostasis and cancer development. These molecules present opposite effects according to tumor type or cancer model. Studies in this area may contribute to unveil glycosaminoglycan activities on cell dynamics during breast cancer exploring these polysaccharides as antitumor agents. Heparanase is a potent tumor modulator due to its protumorigenic, proangiogenic, and prometastatic activities. Several lines of evidence indicate that heparanase is upregulated in all human sarcomas and carcinomas. Heparanase seems to be related to several aspects regulating the potential of breast cancer metastasis. Due to its multiple roles, heparanase is seen as a target in cancer treatment. We will describe recent findings on the function of HSPGs and heparanase in breast cancer behavior and progression.

## 1. Introduction

Breast cancer is defined as a cancer that originates in tissues of the breast, more frequently in the ducts and lobules. It prevails in women, although male breast cancer is also observed. In 2013, the National Cancer Institute estimates 232,340 (female) and 2,240 (male) new cases of breast cancer in the United States with 39,620 female and 410 male deaths, respectively.

Ductal carcinoma in situ (DCIS) is the most common type of noninvasive breast cancer. This cancer begins in cells of the milk ducts. Approximately, 7 out of 10 women with breast cancer have ductal carcinoma. The most common treatment for DCIS consists of lumpectomy, a procedure where most of the breast is conserved, followed by radiation therapy. However, in some cases, removal of the breast or mastectomy is recommended. Alternatively, when DCIS is hormone receptor-positive, hormonal therapy to lower the amount of estrogen in the body is recommended after surgery. 

Invasive ductal carcinoma (IDC) is the most common type of invasive breast cancer, representing about 80% of all breast cancers. Two broad categories are employed for treating IDC: local treatment, consisting of surgery and radiation, or systemic treatment, consisting of chemotherapy, hormonal therapy and targeted therapies.

Invasive lobular carcinoma (ILC) is the second most common type of breast cancer following IDC. Each year, according to the American Cancer Society, more than 180,000 women in the United States are diagnosed with ILC. Other women have a mixture of ductal and lobular type or they have a less common type of breast cancer.

Similar to IDC, treatment for ILC consists of local surgery and radiation, or systemic chemotherapy, hormonal therapy, and targeted therapies. Both in IDC and ILC, the chemotherapy usually follows similar drugs: doxorubicin, epirubicin, cyclophosphamide, docetaxel, paclitaxel, capecitabine, ixabepilone, methotrexate, and 5-FU. 

Breast cancer is a complex, heterogeneous, tissue-specific disease. It is one of the most important malignancies affecting women and the leading cause of cancer-related deaths worldwide. During the last 30 years, several basic and clinical studies to understand the biology of breast tumor cells and to treat breast cancer have improved survival rates of patients [1]. These studies have been historically focused on genetic components involved in tumor progression and tumor microenvironment. The latter is a complex entity composed of several cell types immersed in extracellular matrix (ECM) composed by molecules, such as laminin, fibronectin, collagen, proteoglycans, and matrix metalloproteinases and heparanase. 

Heparan sulfate proteoglycans (HSPG), predominantly found in the ECM and cell surface, are involved in cell signaling, cell adhesion, extracellular matrix assembly, and growth factors storage [2]. As a central molecule, HSPG can regulate cell behavior and tumor progression. Indeed, it has been reported that high levels of the cell surface HSPG syndecan are associated with an aggressive phenotype and poor prognosis in breast cancer patients [3]. Glypican, a GPI-anchored cell surface HSPG, is also highly expressed in human breast cancer cells and regulates mitogenic cell response to heparin-binding growth factors [4].

Despite the central role of HSPG in eukaryotic organisms, there is only one enzyme able to act upon heparan sulfate (HS) chains, named heparanase. This enzyme is an endo-*β*-D-glucuronidase, which cleaves heparan sulfate in fragments of 4 to 7 kDa [5]. In order to display enzymatic activity, heparanase needs to be activated by cleavage, yielding a heterodimer of 8 and 50 kDa subunits [6]. Heparanase is more expressed in tumors, compared to normal tissues [7]. Moreover, this glucuronidase regulates angiogenesis, metastasis, and tumor growth [8], contributing to an aggressive behavior of breast tumor cells and to poor prognosis in breast cancer patients [9]. In this review, we will describe recent findings on the function of HSPGs and heparanase in breast cancer behavior and progression. 

## 2. Heparan Sulfate in Breast Cancer

**Figure 1:**
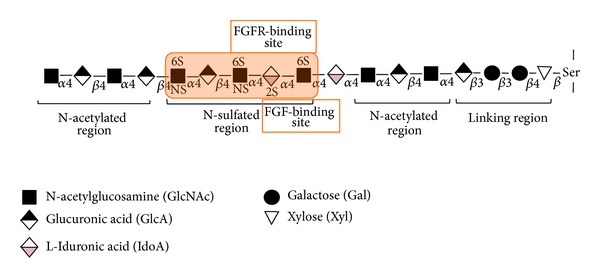
Heparan sulfate structure. Heparan sulfate glycosaminoglycan is linked to specific serine residues on heparan sulfate proteoglycans by a tetrasaccharide sequence of glucuronic acid, galactose, galactose, and xylose. The HS chains contain clusters of N-acetylated unmodified domains and N-sulfated modified domains. Specific sequences in the N-domains bind to different growth factors and their receptors, for example, fibroblast growth factor-2 (FGF-2) and its receptor (FGFR).

Heparan sulfate is a linear glycosaminoglycan composed by repeating disaccharide unities of uronic acid (glucuronic acid or iduronic acid), 1,4 linked to glucosamine. Sulfate substitutions can occur at carbon 2 of the iduronic acid units, and at carbon 3 and/or 6 of the glucosamine, which can also be N-deacetylated followed by N-sulfation (Figure 1). HS has been described to participate in numerous processes during cancer progression [10]. The main importance of this glycosaminoglycan in tumor growth is its ability to bind key growth factors and stromal signaling molecules that activate tumor cells, influencing signaling, cell-cell interactions, uncontrolled proliferation, microenvironment modulation, and migration. HS is synthesized onto different protein cores, forming cell surface proteoglycans (syndecan and glypican) or extracellular matrix proteoglycans (perlecan, agrin, collagen XVIII) [2]. During cancer, HSPGs are usually differentially expressed in comparison to healthy tissue and cells, and drugs that affect HSPGs expression commonly affect other malignant aspects of the tumor [11, 12].

### 2.1. Mammary Development and Heparan Sulfate

The embryonic development of mammary gland becomes evident during midgestation, when placodes are formed and invaginate, following the milk line, to form buds. Bud formation then initiates gland growth, resulting in the structure of a rudimentary gland by the end of the gestational period. Only later, during puberty, mammary gland continues to develop, forming branches [13]. 

Mammary development depends on numerous cellular events such as cell-cell and cell-matrix interactions. In these events, ECM and cell surface proteoglycans play a central role. The HSPGs, syndecans, perlecans, and glypicans, for example, are frequently present during mammary formation, [14–19]. The activity of these HSPGs may rely on the core protein or on the HS glycan chain. It has been described that HS chains play an important role during branching morphogenesis. Garner et al. showed that lack of HS primary central core, after deletion of Ext1, the enzyme responsible for building up the central core of 1,4-linked uronic acid (D-glucuronic acid or L-iduronic acid) and D-glucosamine, results in highly defective mammary development [20]. It was also shown that N-sulfation is very important for mammary gland development, since N-deacetylase/N-sulfotransferase (NDST) 1 and 2 depletion results in hyperbranching [21], and specific deletion of NDST1 inhibits lobuloalveolar expansion [22]. Additionally, HSPGs expression seems to fluctuate along menstrual cycle [23], an indication that these molecules also have a role in the maintenance of the adult tissue.

### 2.2. Heparan Sulfate in Breast Cancer

HSPGs have been described as tumor biomarkers [24–29], being upregulated in aggressive phenotype, or downregulated when tumorigenesis is attenuated in tumor tissues [30–33]. For example, syndecan-2 and syndecan-4, as well as glypican-1, are overexpressed in breast cancer cell lines, compared to normal mammary cells [34], and are mediators of growth factor signaling. EGF/IGF-mediated upregulation of HSPG gene expression enhances breast tumor cell proliferation [35]. Recently, a direct relationship between growth factor signaling and estrogen receptors (ER) has been shown. Breast cancer cells that express ER can be directly stimulated via estrogen, or indirectly stimulated via epidermal growth factor receptor (EGFR) or insulin growth factor receptor (IGFR). Activation of these pathways is crucial for tumor establishment and development and lead to specific modulation of HSPGs, such as syndecans-2 and -4 and glypican-1, in addition to other ECM-modulating molecules [36, 37]. 

 Syndecan-1 has been thoroughly described as a protumorigenic agent during breast cancer development [38–41], especially in the shed form. Glypicans display different activities in tumor development, while glypican-1 overexpression in tumor cells triggers mitogenic response [4], absence of glypican-3 expression in breast cancer cell lines inhibits mammary tumorigenesis [19]. The work by Buchanan et al. showed that glyplican-3 reexpression in murine mammary adenocarcinoma inhibits the PI3/Akt antiapoptotic pathway [42]. Glypican-3 is overexpressed in hepatocellular carcinoma (HCC) and a valued serum diagnostic marker of the disease [43]. More recently, glypican-3 also became a potential and reliable biomarker for predicting tumor recurrence and overall survival in HCC patients after curative resection [44]. Similar to glypican-3, perlecan has also been shown to be downregulated or absent in breast tumors [30, 45].

HSPGs at the surface of breast cancer cells act as growth factor coreceptors, especially for FGF-2 [46, 47]. HS chains bind to different FGFs with reasonable specificity according to its sulfation pattern. For example, a trisaccharide sequence of 2-O-sulfated iduronic acid flanked by N-sulfated glucosamines has recently been shown to be the minimum binding motif and N-sulfation was found to be critical for the binding of HS to FGF-2 [48] (Figure 1). This provides useful information for further development of more potent compounds towards FGF-2 binding, which can have potential applications in wound healing and anticancer therapy.

Disruption of HS sulfation inhibits tumor cell migration, while addition of specific exogenous HS recovers tumor behavior [32]. Growth factor binding specificity leads to different responses according to cell status and the type of HS chain presented by the cells [49–52] and for that function, a balance between cell surface and shed HSPGs, such as syndecan, is crucial. Early reports on syndecan-1 depletion in normal mammary epithelial cells show that these cells acquire a mesenchymal phenotype, losing epithelial markers and presenting a fibroblastoid morphology [53]. The work by Nikolova et al. shows that expression of a shed form of syndecan-1 in MCF-7 cells lowers proliferation rate and enhances migration features, such as Matrigel invasiveness potential. On the other hand, expression of an uncleavable membrane-bound syndecan-1 accelerates proliferation and inhibits matrigel invasiveness [54]. In addition, it has been shown that expression of active heparanase also promotes shedding of syndecan-1, and heparitinase treatment substitutes heparanase activity, indicating that breaking HS chains promotes syndecan shedding [55, 56]. Syndecan-1 shedding also influences FGF-2 signaling via glypican-1, as early shedding promotes glypican-1-dependent FGF-2 signaling. However, maintenance of syndecan-1 on the cell surface promotes glypican-1 independent FGF-2 signaling [57].

The use of HS-related compounds as antitumor agents has been reported. For example, low anticoagulant heparin reduces P-selectin adhesion in breast cancer cells, leading to attenuation of metastasis [58]. HS and heparin oligosaccharides have also been tested to inhibit HS-dependent tumor behavior. The inhibitory activity is achieved during different aspects of cancer development, such as migration, metastasis formation, and tumor growth [59]. In addition, a protective role of glypican-3 has been reported by Peters et al., showing that expression of rat Glypican-3, on mouse breast cancer cell line LM3, inhibited aggressive behavior by maintaining adequate levels of protective molecules [60]. On the other hand, compounds that interfere with HSPGs expression also have a positive effect on tumor inhibition. The bisphosphonate zoledronate, for example, is able to downregulate syndecans-1 and -2 and glypican-1, while upregulating syndecan-4 in a cell line model [11]. This effect is accompanied by inhibition of growth, invasion, and adhesion of tumor cells, in addition to inhibition of osteoclast activation in a cellular model of breast cancer bone metastasis [61].

### 2.3. Heparan Sulfate 6-O-Endosulfatases in Breast Cancer

HS 6-O-endosulfatases, also known as Sulfs, are deeply involved in the metastatic behavior of breast tumor cells [62]. It has been shown that the two isoforms Sulf1 and Sulf2 seem to present different activities during tumor growth. Lai et al. showed that Sulf1 expression is low in breast and ovarian cancer, and induction of enzyme expression inhibits tumor behavior in cells [63]. Narita et al. showed that high expression of Sulf1 in tumor cells fails to develop vessels, leading to marked necrosis and apoptosis, and this probably occurs due to the inability of tumor endothelial cells to bind FGF-2 [64]. On the other hand, Sulf2 has been shown to be proangiogenic [65], and depletion of this enzyme leads to reduced tumor size [66]. Bret et al. showed SULF1 and SULF2 mRNA overexpression in breast cancer cohorts from different parts of the world [67]. On the other hand, Peterson et al. reported that transfection of human Sulf1 and Sulf2 in MDA-MB-231, a human breast cancer cell line, affected xenografts growth, while a single injection of purified sulfotransferases did not have the same effect [68]. Overall, Sulfs1 and 2 seem to have opposite effects, but compensation mechanisms on tissue response to tumor still need to be explored in order to unveil the activities of Sulfs on breast cancer development.

### 2.4. Heparan Sulfate in Breast Cancer Environment

Other cell types, such as endothelial, immune cells, and fibroblast, surround breast cancer cells. HS-dependent crosstalking between these cell types and tumor cells also plays an important role during breast cancer development. Lines of evidence suggest that conditioned media from endothelial cells inhibit breast cancer cells invasiveness, and depletion of perlecan may disrupt this ability [69]. Also, lymphocytes derived from breast cancer patients affect healthy lymphocytes, turning them into tumor-inducing cells via heparanase expression [70]. Fibroblasts derived from breast carcinoma tissue produce MT1-MMP, which leads to syndecan-1 shedding from tumor cells, an important step in tumor invasion, while fibroblasts derived from healthy mammary tissue do not possess the same effect [71]. 

### 2.5. Other Sulfated Glycosaminoglycans in Breast Cancer

Other sulfated glycosaminoglycans, such as chondroitin sulfate (CS) and dermatan sulfate (DS), are also involved in mammary gland development [23] and may, consequently, be involved in breast cancer development. The activity of CS in breast cancer is still contradictory. In one hand, CS overexpression by tumor cells is associated with a poor prognostic phenotype. On the other hand, downregulation of the glycosaminoglycan is described in aggressive tumors [72]. CS has been reported as an aggressive tumor molecule, showing increased levels in tumor tissue samples compared to normal tissue [73]. The role of CS during metastasis still needs to be explored. CS participates in P-selectin binding in a cell line model, which suggests that this glycosaminoglycan is involved in the metastatic process [74, 75]. Controversially, chondroitinase ABC treatment in tumors triggers metastasis [76]. It is still unclear whether CS is relevant to breast cancer development; however, its expression and overall activity seem to correlate with a more aggressive tumor phenotype and, consequently, poor prognosis. 

DS chains are reduced in breast tumor samples compared to healthy neighbor tissue, both glycan chains and core proteins had their levels altered [77–79]. Nevertheless, DSPGs expression were described to be increased in breast cancer fibroadenoma compared to healthy tissue [80], and, although the DSPG decorin was present in both healthy and tumor tissue, versican was exclusively detected in tumor samples.

**Figure 2:**
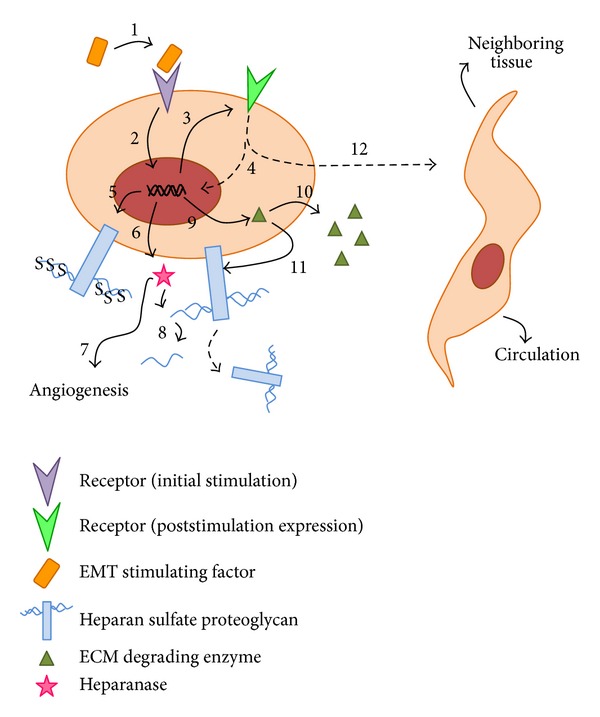
Model of how Heparan sulfate proteoglycans and heparanase participate in the epithelial-to-mesenchymal transition of a breast cancer cell. When a specific factor, such as TGF-*β*, stimulates its receptor on the tumor cell surface (1), the signaling cascade triggers transcriptional changes (2) that lead to a differential expression of specific receptors (3), which will allow the tumor cell to become responsive to other available factors that will culminate in the transition from an epithelial to a mesenchymal state (4). During this process, these transcriptional changes also lead to higher degree of sulfation of heparan sulfate chains (5), enhancing the cell ability to bind more extracellular molecules. Also, heparanase expression takes place (6), enhancing tumor angiogenesis (7) and degrading heparan sulfate chains (8) that will no longer be internalized, staying in the extracellular matrix bound with factors that also cooperate in the epithelial-to-mesenchymal transition process. Expression of extracellular matrix-degrading enzymes (9), such as metalloproteinases, promote extracellular matrix degradation (10) and heparan sulfate proteoglycans shedding (11). These processes altogether culminate in a complete transformation of an epithelial tumor cell into a mesenchymal phenotype (12) able to invade the neighboring tissue and circulation.

In conclusion, significant data have been generated over decades correlating HSPGs expression and modulation with EMT and metastasis (Figure 2). The literature shows that HS accompanied by its glycosaminoglycan counterparts regulate tissue homeostasis and cancer development. In different circumstances, these molecules present opposite effects according to tumor type or cancer development model. Further studies in this area may contribute to unveiling sulfated glycosaminoglycan activities on cell dynamics during breast cancer and explore these polysaccharides as antitumor agents.

## 3. Heparanase and Its Regulatory Function on Breast Cancer

Heparanase is a potent modulator of tumor behavior due to its protumorigenic, proangiogenic, and prometastatic activities. Several lines of evidence indicate that heparanase is upregulated in all human sarcomas and carcinomas [81] and occurs at elevated levels in body fluids of breast cancer patients [82]. Indeed, evaluation of heparanase expression in breast cancer cells reveals that more aggressive lines, such as MDA-MB-231 and MDA-MB-435, present high levels of heparanase, whereas MCF-7, a nonmetastatic and poorly invasive luminal breast cancer cell, presents low levels of the enzyme [7]. The enzymatic activity of heparanase has been assessed, and the results show that aggressive lines present high activity, while nonmetastatic cells present low or no activity. Moreover, in an orthotopic model of MCF-7 cells transfected with the HPSE gene, tumors are able to grow faster, presenting increased vascularization and a higher degree of vessel maturation in comparison with tumors formed by control cells [8]. On the other hand, heparanase gene silencing reduces invasive ability of MDA-MB-435 [83].

### 3.1. Heparanase Function in Metastasis

Heparanase seems to be related to several aspects that regulate the potential of breast cancer metastasis. It has been shown that breast cancer cells from brain metastatic sites exhibited high levels of heparanase, compared to parental cells [84]. In this case, metastatic cells respond to EGF by phosphorylation of EGF and HER-2 receptors and by increasing heparanase levels. EGF also induces heparanase translocation to the nucleus [85]. DNA topoisomerase I, a key player during DNA replication, is regulated by nuclear heparanase, thus affecting cell proliferation of breast cancer cells in brain metastases [84]. Moreover, colocalization of heparanase and DNA topoisomerase I in the nucleus was found only in slices obtained from metastatic brain that overexpress HER2, confirming the idea that heparanase is a downstream molecule arisen from HER2-induced signaling [84]. Heparanase has also been shown to play a role in bone metastasis. Tumors formed by a variant of bone-colonizing MDA-MB-231 cells, which overexpress heparanase, are capable of inducing bone reabsorption without detectable bone metastasis, indicating that heparanase may have a role prior to the establishment of macrometastasis [86].

### 3.2. Modulation of Heparanase Expression

Several factors regulate heparanase gene expression in cancer cells. Early growth response gene 1 (EGR-1) is a zinc-finger transcription factor that plays dual role in tumor biology [85]. In breast tumor cells, EGR-1 binds to heparanase promoter and induces its activity in a dose-dependent manner [87]. Heparanase gene regulation is also modulated by estrogen, which is an important risk factor for breast cancer. Estrogen exposure enhances heparanase promoter activity in MCF-7 cells, and this can be associated with four estrogen response elements in the heparanase promoter [8]. The importance of these data was confirmed by breast cancer tissue array, which demonstrates a correlation between estrogen receptor (ER) and heparanase expression [88]. Moreover, this work reveals that Tamoxifen, a common drug used to treat breast cancer (ER+) patients, induces heparanase expression in MCF-7 and T47D cells.

Other transcriptional factors can modulate heparanase expression in breast carcinoma, such as the tumor suppressor p53. Mutation in tumor suppressor p53 gene is associated with several human tumors, altering cell behavior to favor tumor progression. Indeed, tumor suppressor p53 mutations are more common in high-grade ductal carcinoma in situ (DCIS) than in low-grade DCIS [89]. Additionally, p53 binding to heparanase promoter exerts inhibitory actions, whereas mutant p53 is not able to induce the same effect [90]. Also, ETS transcription factors, ETS1 and ETS2, regulate heparanase expression by binding to four different sites in the regulatory sequence of the heparanase gene [91].

### 3.3. Heparanase as an Inducer of Angiogenesis

Angiogenesis is a crucial step of cancer progression in a number of tumor types, including breast cancer. Heparanase seems to contribute to angiogenic responses by promoting cleavage of HS chains, releasing growth factors, such as bFGF and VEGF [81, 92, 93], to bind to endothelial cells. In addition, heparanase can promote VEGF gene regulation in a variety of cells. It was demonstrated that VEGF is upregulated in MDA-MB-435 human breast carcinoma cells overexpressing heparanase. Increase in VEGF expression is mediated via Src activation, and, although p38 phosphorylation is involved, it is not essential [93]. It was also shown that conditioned medium from heparanase-overexpressing cells sustain endothelial cell proliferation. This effect is inhibited by the presence of soluble VEGF receptor, suggesting that VEGF secreted by heparanase-overexpressing cells is involved [93]. Moreover, VEGF upregulation seems to be independent of heparanase enzymatic activity. The enzyme cyclooxygenase-2 (COX-2) triggers heparanase-dependent angiogenic response. COX-2 localization in breast cancer specimens was similar to heparanase and there is a correlation between heparanase and COX-2 expression, which is evident in invasive breast cancer [94]. This work also shows that heparanase expression is related to higher incidence of metastasis and the size of the primary tumor.

### 3.4. Heparanase and miRNA-1258 in Breast Cancer

It has been recently reported that heparanase expression and activity in brain metastatic breast cancer cell (MDA-MB-231) can be inhibited by the microRNA miR-1258 [91]. According to this idea, it was shown that miR-1258 and heparanase levels are inversely correlated in breast carcinoma [95]. Thus, invasive ductal carcinoma and malignant cells showed attenuated expression of miR-1258. Moreover, western blot analysis indicates that miR-1258 inhibits phosphorylation and expression of heparanase-related proteins AKT, EGFR, MMP-9, and COX-2, resulting in decrease of breast cancer brain metastasis [95].

### 3.5. Heparanase Inhibitors

Taking into account the involvement of heparanase in breast cancer, several heparanase inhibitors have been developed in order to affect tumor growth and bulk angiogenesis [96, 97]. Progen Pharmaceuticals Company (Brisbane, QLD, Australia) designed a compound called PG545. This drug is a synthetic HS mimetic, a sulfated tetrasaccharide that decreases tumor growth in MDA-MB-231 xenograft and inhibits angiogenesis *in vivo* [96]. Recently, it was shown that PG545 blunts orthotopic tumor growth and inhibits lung spontaneous metastasis, contributing to overall survival. Importantly, PG545 treatment reduced heparanase expression in the primary tumor and at metastatic foci [97]. 

PI-88 (Phosphomannopentaose sulfate) is a heparanase inhibitor that is in clinical trials (phase II). Prostate cancer treatment with PI-88 in association with docetaxel decreases prostate specific antigen (PSA) response in 70% of patients (Clinical Trials, ID: NCT00268593). PI-88 was already employed in animal models to treat breast cancer [98]. In this context, it was revealed that PI-88 decreases tumor growth rate of highly invasive rat mammary adenocarcinoma, 13762 MAT cells, inhibits metastasis as well as tumoral vascularity. Laminarin sulfate, a linear polymer (*β*-1,3 glucan) sulfated at 2 and 6 position, known as heparanase inhibitor, inhibited lung colonization of 13672 MAT mammary-adenocarcinoma cells [99]. 

Due to its multiple roles in cancer progression, heparanase is seen as a potential target in cancer treatment. Besides inhibitors and antibodies against heparanase, vaccines have been developed. Two reasons explain why heparanase is being seen as a universal tumor-associated antigen: heparanase is expressed in several types of advanced tumors, and dendritic cell expressing heparanase are able to elicit heparanase-specific cytotoxicity T lymphocytes (CTL) against tumor cells [100, 101]. It was shown that vaccines made of heparanase multiple antigenic peptides induce CTL response in heparanase overexpressing MCF-7 cells [102]. Altogether, these data point to heparanase as a good target molecule to break breast cancer progression.

## 4. Future Directions

**Figure 3:**
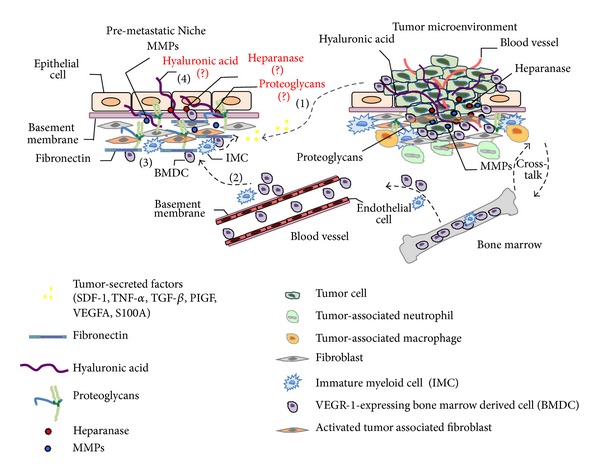
Cellular and molecular composition of premetastatic niche and metastatic microenvironment. premetastatic niche formation initiates by release of soluble factors, such as, VEGFA, TGF-beta, placental growth factor (PLGF), inflammatory chemokines S100, and Serum Amiloyd A3 (SAA3), as well as stromal-derived growth factor 1 (SDF1) by the primary tumor (1). As a result, bone marrow-derived hematopoietic progenitor cells (HPC) and immature myeloid cells are recruited to the premetastatic niche (2). Then, these bone marrow-derived cells start to populate the premetastatic niche with potent modified factors, such as tumor necrosis factor-*α* (TNF*α*), matrix metalloproteinase 9 (MMP9), and TGF*β*, leading to stimulation of stromal cells that in turn modulate the extracellular matrix of the premetastatic microenvironment (3). For example, modified factors-mediated fibroblast activation initiates secretion of fibronectin, which constitutes an important adhesion protein in the niche. Additionally, other important extracellular matrix components such as hyaluronic acid, proteoglycans, glycosaminoglycan-modified enzymes, like heparanase and sulfatases are likely to be present (4), but it is yet to be confirmed and constitutes a new interesting area of research involving the premetastatic niche. Mature tumor microenvironment is composed by tumor cells, blood vessels, bone marrow-derived cells, proteoglycans, MMPs, hyaluronic acid, stromal cells, such as fibroblasts and several recruited cells like neutrophils, and macrophages. These cells, secrete several growth factors and cytokines that can drive epithelial to mesenchymal transition-mediated migration and invasion of tumor cells.

In addition to cancer genomics, host immunology, cell biology to develop less toxic treatments, and narrowing cancer therapy to individual patients, cancer research, including breast cancer, requires a multidisciplinary approach to study different aspects of the tumor biology. This includes tumor microenvironment, extracellular matrix components, and extracellular matrix-associated proteases, such as MMPs, sulfatases, and glycosidases. The knowledge generated in these basic studies should work in parallel with that generated in clinical studies to allow the development of new protocols. For example, nonanticoagulant heparin and heparan sulfate mimetics, as well as heparanase and sulfatase inhibitors, could be used as adjuvant therapies along with other chemotherapeutic protocols. Additionally, the concept of a premetastatic niche, created by evolutionary mechanisms during cancer progression, should be added to the overall picture (for review, see [103]) (Figure 3). This premetastatic microenvironment is constructed by signaling molecules (cytokines, chemokines, and exosomes), secreted by recruited bone marrow derived cells, and as activated resident cells such as fibroblast. As the premetastatic niche matures, as a result of an intense tissue remodeling, clone expansion and tumor promotion occur, leading to the establishment of metastatic focus. Therefore, the extracellular matrix composition of the premetastatic niche, with the probable occurrence of proteoglycans, hyaluronic acid, proteases, and glycosidases, should be an area of intense investigation in the future.
